# Recovery Infectious Enterovirus 71 by Bac-to-Bac Expression System *in vitro* and *in vivo*

**DOI:** 10.3389/fmicb.2022.825111

**Published:** 2022-02-25

**Authors:** Baojing Lu, Qi Tang, Qianyun Wang, Xuejuan Liu, Hui Peng, Binbin Zhu, Li Xie, Zeng Li, Hanzhong Wang, Zhenhua Zheng, Linding Wang, Bao Li

**Affiliations:** ^1^The Key Laboratory of Microbiology and Parasitology of Anhui Province, The Key Laboratory of Zoonoses of High Institutions in Anhui, Department of Microbiology and Parasitology, School of Basic Medical Sciences, Anhui Medical University, Hefei, China; ^2^Inflammation and Immune Mediated Diseases Laboratory of Anhui Province, Hefei, China; ^3^Department of Tuberculosis Prevention, Shenzhen Center for Chronic Disease Control, Shenzhen, China; ^4^CAS Key Laboratory of Special Pathogens and Biosafety, Center for Emerging Infectious Diseases, Wuhan Institute of Virology, Chinese Academy of Sciences, Wuhan, China; ^5^The Comprehensive Lab, School of Basic Medical Science, Anhui Medical University, Hefei, China

**Keywords:** enterovirus 71, recovery, baculovirus, infectious complementary deoxyribonucleic acid (cDNA) clone, *in vitro* and *in vivo*

## Abstract

Enterovirus 71 (EV71) is one of the most important etiological agents for hand–foot–mouth disease. Compared with coxsackievirus A16 infection, EV71 infection is often associated with severe central nervous system complications, such as encephalitis, encephalomyelitis, and acute flaccid paralysis in infants and young children. In this study, we constructed a recombinant baculovirus with T7 ribonucleic acid polymerase under the control of a cytomegalovirus promoter and simultaneously engineered the T7 promoter upstream of a full-length EV71 complementary deoxyribonucleic acid. After transduction into mammalian cells, typical cytopathic effects (CPEs) and VP1 signals were detected in cells transfected with recombinant baculovirus. Additionally, viral particles located in the cytoplasm of human rhabdomyosarcoma cells (Rd) and Vero cells were observed by electron microscope, indicating that EV71 was recovered using a Bac-to-Bac expression system *in vitro*. After four passages, the rescued virus had a growth curve and plaque morphology similar to those of the parental virus. Furthermore, the *Vp1* gene and the protein from the mouse brain were detected by reverse transcription polymerase chain reaction and immunohistochemistry after intracerebral injection of purified recombinant baculovirus. Typical CPEs were observed after inoculation of the supernatant from mouse brain to Rd cells, revealing a reconstruction of EV71 *in vivo*. Thus, we established a new approach to rescue EV71 based on a baculovirus expression system *in vitro* and *in vivo*, which may provide a safe and convenient platform for fundamental research and a strategy to rescue viruses that currently lack suitable cell culture and animal models.

## Introduction

Enterovirus 71 (EV71) belongs to the *Enterovirus* genus of the *Picornaviridae* family, which is a non-enveloped, positive, single-stranded ribonucleic acid virus, that is a common cause of hand–foot–mouth disease (HFMD) in young children (Mao et al., 2016), but occasionally it can also lead to severe diseases such as aseptic meningitis, poliomyelitis-like paralysis, and possibly fatal encephalitis (Solomon et al., 2010). Because of consecutive epidemics over the past years in the Asia-Pacific region including Singapore, Malaysia, Taiwan, and mainland China, this condition has attracted immense concern in global heath (Solomon et al., 2010). Although major efforts have been made to elucidate the molecular mechanisms, virus–host interactions, and immune evasion of EV71 infection, no approved antivirals and limited vaccine and animal models for EV71 have been developed (Mao et al., 2016; Kim et al., 2019; Wang et al., 2021).

Reverse genetics systems are one of the most important and powerful tools to study the molecular biology of viruses (Yu et al., 2019). With the outbreak of emerging viruses, such as SARS-CoV-2, MERS-CoV, or Ebola virus, this system will be important for functional genomics research and virus prevention (Cockrell et al., 2017; Cross and Geisbert, 2019; Thi Nhu Thao et al., 2020). A full-length cDNA clone was always used for the recovery of RNA viruses (Boyer and Haenni, 1994), and the first infectious RNA virus was successfully isolated from cDNA to generate poliovirus in 1981 (Racaniello and Baltimore, 1981). Since then, most positive-strand RNA viruses, such as hepatitis A virus, coxsackievirus B6, and norovirus, have been recovered based on the reverse genetics system (Tellier et al., 1996; Martino et al., 1999; Todd and Tripp, 2019). Arita et al. (2005) constructed an infectious clone of BrCr to explore the neurovirulence site of EV71, which was the first study of EV71 rescue to the best of our knowledge. Then various groups constructed infectious clones of EV71 using different strategies that showed potential advantages for gene mutation, animal model application, and vaccine production of rescue virus (Han et al., 2010; Meng et al., 2012; Zhang et al., 2020).

Various reverse genetics strategies are currently applied for virus recovery. Direct transfection of *in vitro*-synthesized RNA transcripts is a traditional and effective method for virus recovery especially for RNA viruses whose genome is smaller than 15 kb. Alternatively, a recombinant vaccinia virus with a T7 RNA polymerase (VacT7) system has been widely used for virus reconstruction, such as SARS-CoV (van den Worm et al., 2012), vesicular stomatitis virus (Lawson et al., 1995), and nodavirus (Lawson et al., 1995) because of its easy handling, higher expression level, and specificity. In these systems, the full-length cDNA molecule is placed downstream of the T7 or SP6 promoter, and the entire genomic RNA is either synthesized *in vitro* and the transcripts introduced into host cells, or the RNA is synthesized by the use of a T7 RNA polymerase-expressing helper virus. Therefore, the transformation efficiency, stability of RNA, and enzyme activity of T7 RNA polymerase are possible factors affecting the recovery of viruses *in vitro*.

The baculovirus expression vector is recognized as a useful viral vector not only for abundant expression of foreign proteins in insect cells but also for gene delivery into mammalian cells (Jarvis, 2009). Baculoviruses have many attractive features, such as easy manipulation, a large capacity for foreign DNAs, and a wide vertebrate host range (Jarvis, 2009). An additional advantage of the baculovirus expression system is that a single recombinant virus can express more than four foreign genes, and this system has also been widely used for drug screening, vaccine development, and gene therapy (Roldão et al., 2011). It is also an effective vector for constructing infectious clones. A T7 RNA polymerase recombinant baculovirus was explored in recovering poliovirus indicating possible recovery of infectious virus from cDNA clones (Yap et al., 1997). Delaney also applied recombinant HBV baculovirus to deliver the HBV genome to HepG2 cells (Delaney and Isom, 1998), and Lucifora produced infectious HBV by delivering a novel recombinant baculovirus to HepG2 cells in 2008 (Lucifora et al., 2008). To date, there has been no research on EV71 recovery by baculovirus expression systems.

In this study, we developed a new strategy for the recovery of EV71 by a baculovirus expression system. We constructed one recombinant baculovirus synthesizing T7 RNA polymerase under the control of the cytomegalovirus (CMV) promoter and simultaneously engineered the T7 promoter upstream of a full-length cDNA clone of BrCr for transcription. After transduction into mammalian cells and intracerebral injection of the baculovirus into suckling mice intracerebrally, infectious EV71 was recovered *in vitro* and *in vivo*. Our baculovirus-based reverse genetics approach may provide a new strategy for EV71 rescue and apply it in molecular biology and antiviral research.

## Materials and Methods

### Cells, Viruses, and Antibodies

*Spodoptera frugiperda* (Sf9) cells were cultured in Grace’s medium (Invitrogen, Carlsbad, CA, United States) supplemented with 10% (v/v) fetal bovine serum (FBS, Gibco) at 27°C; Vero and BHK cells were cultured in Dulbecco’s modified Eagle’s medium (DMEM; Life Technologies, Grand Island, NY, United States) supplemented with 10% heat-inactivated FBS at 37°C with 5% CO_2_. Human rhabdomyosarcoma cells (Rd) and human neuroblastoma cells (Sk) were grown in minimum essential medium (MEM; Life Technologies, Grand Island, NY, United States) containing 10% FBS at 37°C with 5% CO_2_. The EV71 BrCr strain was provided by the Institute of Medical Biology, Chinese Academy of Medical Sciences. The polyclonal antibody against EV71 VP1 for Western blotting was prepared by our laboratory, and the monoclonal antibody used for immunohistochemistry was purchased from NZK Biotech (Wuhan, China). β-actin antibody was purchased from Proteintech (Wuhan, China).

### Recombinant Bacmid Construction

The donor plasmid pFB-CMV-Ef1-α was derived from pFastBacTM Dual (Invitrogen, Carlsbad, CA, United States) and replaced the p10 promoter (Pp10) and polyhedrin promoter (PpH) with cytomegalovirus and the Ef1-α promoter (Bai et al., 2008). The infectious cDNA clone pEV71-BrCr-TR (AB204852) used for PCR was kindly supplied by Dr. Arita (Arita et al., 2005). The pEV71-GFP-BrCr cDNA clone, in which a GFP gene was inserted between the 5′ UTR and N-terminus of the VP4 gene of pEV71-BrCr-TR was constructed as previously described (Shang et al., 2013). The T7 RNA polymerase gene was amplified by PCR from BL21 (DE3) using the specific primers T7-F: 5′-ATC*GTCGAC*GCCACCATGAACACGATTAACATCG-3′ and T7-R: 5′-CA*AAGCTT*TTACGCGAACGCGAAGTCCGAC-3′ (underlined and italicized sequences are *Sal*I and *Hin*dIII sites, respectively). After sequence confirmation, this gene was cloned into pFB-CMV-EF1-α using the *Sal*I and *Hin*dIII sites and named as pFB-CMV-T7-EF1-α. The cDNA from the 5′ end to the nucleotide position 2,915 with a T7 promoter before the 5′ end of the BrCr genome was amplified using pEV71-BrCr-TR or pEV71-GFP-BrCr as the template, and the primers used were as follows: BrCr-2915-F: 5′-TCA*ATGCAT*GCTCGACTGGCTTATCGAAATTACG-3′ and BrCr-2915-R: 5′-TA*CCCGGG*TGGAACAAACAT-3′ (underlined and italicized sequences were *Nsi*I and *Sma*I sites, respectively). After digestion with *Nsi*I and *Sma*I, the PCR product was cloned into pFB-CMV-T7-EF1-α. The rest of the cDNA of BrCr obtained from the pEV71-BrCr-TR was cloned into the above construct following the digestion by *MIu*I and *Sma*I, resulting in the donor bacmids pFB-T7-BrCr and pFB-T7-GFP-BrCr, which contained the full-length cDNA clone of BrCr. All sequences of the intermediate product were validated by sequencing before use in the subsequent cloning step.

### Recombinant Baculovirus Construction, Purification, and Titration

The recombinant viruses (Ac-T7-BrCr, Ac-T7-GFP-BrCr) were generated by transfecting the indicated bacmids into Sf9 cells according to the manufacturer’s instructions (Bac-to-Bac baculovirus expression system, Invitrogen, Carlsbad, CA, United States). The recombinant baculovirus and wild-type virus were collected 60 h after infection, and cell debris was removed by centrifugation at 4,000 × *g* for 20 min. The supernatant was filtered through a 0.45-mm filter (Millipore, Burlington, VT, United States) and subsequently centrifuged at 25,000 rpm at 4°C for 90 min (BeckmanSW28 rotor) in 5 ml of a 30% (w/v) sucrose cushion in TE buffer (Wang et al., 2010). The virion pellet was resuspended in TE buffer, sucrose was removed by ultrafiltration (100 kDa, Millipore, Burlington, VT, United States), and the pellet was suspended in PBS and stored at –80°C. The viral titer was determined by the 50% tissue culture infectious dose (TCID_50_) assay using the Reed–Muench method (Svensson et al., 1999).

### Transduction of Mammalian Cells With the Baculovirus

Mammalian cells were seeded in six-well culture dishes at a concentration of 2 × 10^5^ cells per well. Eight hours after adherence, the medium was removed and replaced with virus (vAc-T7-BrCr, vAc-T7-GFP-BrCr, or wild-type AcMNPV) at a multiplicity of infection (MOI) of 50, and the cells were incubated for 6 h at 37°C. Then, the cells were washed and replaced with 2 ml of fresh medium.

### RNA Isolation and Real-Time Polymerase Chain Reaction Analysis

Total RNA was extracted using TRIzol reagent (Invitrogen, Carlsbad, CA, United States) according to the manufacturer’s protocol. First-strand cDNA was reverse transcribed from 2 μg of total RNA using the PrimeScript™ RT reagent kit with gDNA Eraser (TaKaRa, Dalian, China). Real-time PCR was conducted with SYBR Green Master Mix (Bio-Rad) on a CFX Connect Real-Time PCR Detection System from (Bio-Rad, Hercules, CA, United States). Specific primers used for qRT-PCR were previously described (Zheng et al., 2013), and GAPDH mRNA was measured as a control.

### Western Blot Analysis of Structural Proteins

Cells were harvested and lysed with Western lysis buffer (Beyotime, Shanghai, China). After electrophoresis, proteins were transferred to PVDF membranes (Millipore, Burlington, VT, United States) for 30 min by semidry electrophoresis transfer (Bio-Rad). Then the membranes were blocked in 20 mM Tris–HCl buffer (pH 7.4) containing 37 mM NaCl with 5% BSA (Sigma-Aldrich, St. Louis, MO, United States) at 37°C for 1 h. After that, the film was incubated with polyclonal antibodies against VP1 (diluted 1:1,000 in TBS containing 0.1% Tween 20 and 2% BSA; our lab performed) and β-actin (1:1,000; Proteintech, Wuhan, China) overnight at 4°C. After three washes in TBST for 10 min each time, the membrane was incubated with AP-conjugated secondary antibody (Boster, Wuhan, China) for 2 h at room temperature. Finally, the signals were detected using a BCIP/NBT alkaline phosphatase color development kit (Beyotime, Shanghai, China).

### Electron Microscopy Imaging of Recovered Viruses

Thirty-six hours post-transduction with vAc-T7-BrCr, Vero and Rd cells were fixed at 4°C with 2.5% (W/V) glutaraldehyde in 0.1 mol/L PBS (pH 7.2) overnight. Then the electron microscopy samples were prepared as previously described (Wang et al., 2010) and observed by transmission electron microscopy (FEI Tecnai G2, operated at 200 kV).

### Viral Growth Kinetics

After transducting Vero cells with v-Ac-EV71, a rescued EV71 was obtained, and Rd cells were infected with the virus to amplify and obtain the F4 generation of virus. Rd cells were seeded in 12-well plates (2 × 10^5^ cells per well, and the confluence reached approximately 60% the next day). The cells were separately infected with wild-type EV71 (WT EV71) or rescued EV71 (rEV71) at an MOI of 1. After 2 h of adsorption and washing with PBS twice, the supernatant was replaced with 2 ml of MEM + 10% FBS. Then the supernatant was collected at 6, 12, 16, 24, and 30 h postinfection, and viral titers were quantified by TCID_50_ assay.

### Plaque Assays

Wild-type EV71 or rEV71 with the same TCID_50_ was diluted 1:10 by mixing 80 μl of virus sample with 720 μl of MEM. Two hundred microliters was added to individual wells of 12-well plates containing confluent Rd cells (2 × 10^5^ cells per well, the plates were plated 1 day in advance, and a cell monolayer was formed 24 h later). After infection, the plates were adsorbed for 2 h and washed twice with PBS, and 1.5 ml of 2 × MEM (containing 4% fetal bovine serum) containing 1% agarose was added to individual wells of 12-well plates. Each well was fixed with 1 ml of crystal violet containing formaldehyde and dyed overnight after the plates were incubated at 37°C with 5% CO_2_ for 48 h. The numbers and morphology of plaques were recorded after the overlaying layer was flushed away with running water the next day.

### Mice and Inoculation of Baculovirus

One-day-old ICR mice were obtained from the Centers for Disease Prevention and Control of Hubei Province and maintained under specific pathogen-free (SPF) conditions. The mice were randomly divided into the experimental group and the control group (10 mice each). A total of 1 × 10^7^ TCID_50_ purified recombinant baculoviruses Ac-T7-BrCr and AcMNPV in a volume of 20 μl or PBS was intracerebrally inoculated into mice using a 50-μl Hamilton injector (Hamilton Co., Reno, NV, United States). All experimental procedures were conducted according to the guidance of the Institutional Animal Care and Use Committee of Wuhan Institute of Virology, Chinese Academy of Sciences (No: WIVA07201502).

### Sample Collection and Virus Detection

Brain samples were collected 7 days after inoculation. The samples were washed with sterilized PBS and dissected in half along the coronal line; one half was stored at –80°C, and the other was fixed in 4% paraformaldehyde for immunohistochemistry detection. For the RT-PCR assay, the samples were suspended in TRIzol for RNA extraction. Brain samples suspended in PBS were disrupted by a microelectric tissue homogenizer (Kimble, United States) and sterilized with a 0.22-μm filter for virus isolation in Rd cells.

### Immunohistochemistry Detection and Immunofluorescence Assay

Brain samples were fixed in 4% paraformaldehyde and embedded in paraffin. Sections (5 μm) were sliced by a section cutter (Leica, Wetzlar, Germany) and then immunostained overnight with anti-EV71 mAb (1:500) against VP1 at 4°C. Subsequently, the positive cells were visualized with a PV-6002 HRP-conjugated goat anti-mouse detection kit (Zhongshan, Beijing, China) and recorded by a light microscope. The immunofluorescence assay was carried out as previously reported (Pham et al., 2019; Tang et al., 2020). In short, the infected or non-infected Vero cells were washed by PBS three times, followed by fixing with 4% paraformaldehyde for 15 min and permeabilizing for 15 min with 0.5% Triton X-100. Subsequently, samples were incubated with VP1 antibody (1:200) at 4°C overnight. Finally, the positive cells were visualized with Alexa Fluor 647 Conjugate anti-mouse immunoglobulins (Cell Signaling Technology, Danvers, MA, United States) and DAPI (Beyotime, Shanghai, China) and recorded by a Leica confocal microscope.

## Results

### Transfer Vector Construction and Characterization of Recombinant Baculovirus

**FIGURE 1:**
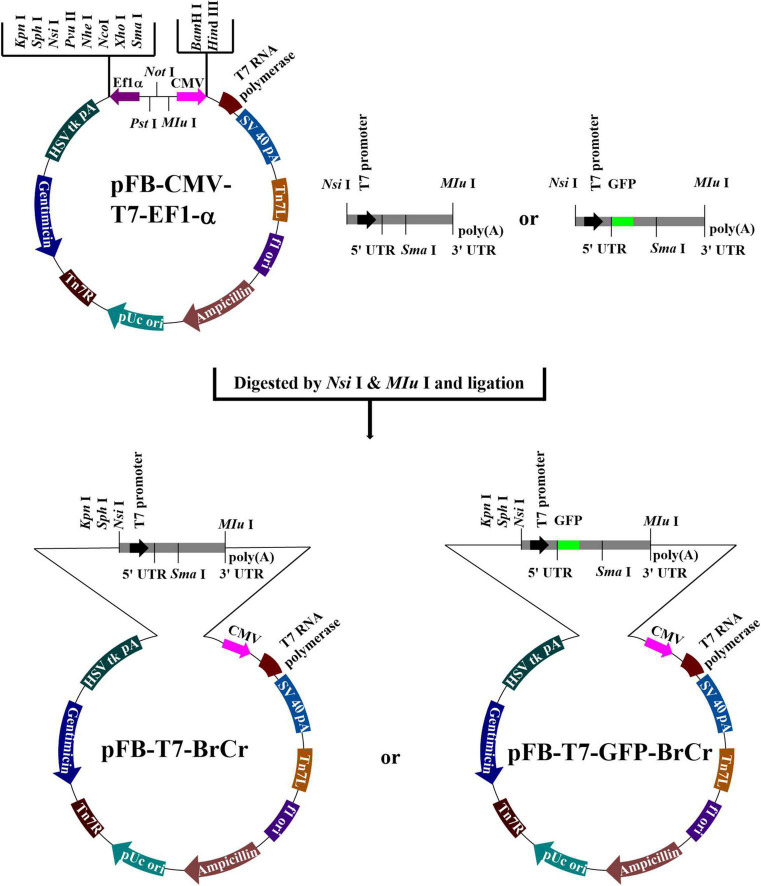
Construction strategy of transfer vectors. The amplified gene of T7 RNA polymerase was inserted into pFB-CMV-EF1-α by *Sal*I and *Hin*dIII. cDNA from the 5′ end to nucleotide position 2,915 with *MIu*I with a T7 promoter before the 5′ end of the BrCr genome was cloned into pFB-CMV-T7-EF1-α by *Nsi*I and *Sma*I, and the rest of the cDNA was cloned by *Sma*I and *MIuI*.

The construction strategy for the transfer vector of pFB-T7-BrCr and pFB-T7-GFP-BrCr is as shown in Figure 1. Then recombinant bacmid DNA was transfected into Sf9 cells to produce recombinant baculovirus virus, and the virus Ac- T7-BrCr, which can express T7 RNA polymerase in mammalian cells and contains the T7 promoter before the genome of BrCr was constructed using a Bac-to-Bac expression system. Furthermore, to observe whether infectious EV71 can be synthesized after transduction, we constructed the baculovirus Ac-T7-GFP-BrCr carrying a GFP gene in the genome of EV71 as previously described (Shang et al., 2013).

### Transduction of Vero Cells With Recombinant Baculovirus

**FIGURE 2:**
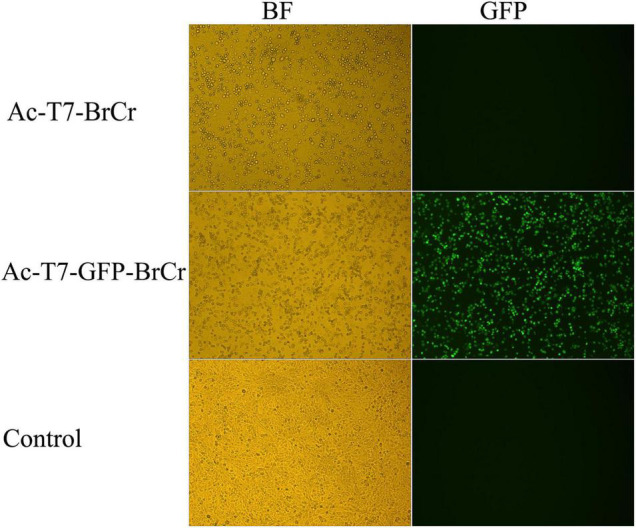
Enterovirus 71 (EV71) reconstruction in Vero cells. Vero cells were transduced with the baculoviruses Ac-pFB-T7-GFP-BrCr-BrCr, Ac-T7-GFP-BrCr, or wild-type AcMNPV. The left and right panels represent the same field of view. The left panels were visualized in bright field, and the right panels were visualized with a fluorescein isothiocyanate (FITC) filter set.

To obtain the recovered infectious virus from the full-length cDNA clone, we transduced Vero cells with the baculoviruses Ac-T7-BrCr, Ac-T7-GFP-BrCr, or AcMNPV. Rescued EV71-induced cytopathic effects (CPEs) and fluorescence appeared 18 h posttransduction, and approximately 80% of the cells were rounded and lost normal cell morphology 48 h posttransduction compared with the controls. This result indicated that EV71 cDNA with a 5′-terminal T7 promoter was efficiently transcribed to generate infectious virus that replicated in permissive Vero cells and showed a specific CPE (Figure 2). At the same time, the culture medium from the transfected cells was collected, and viral titers were determined to be 3.6 × 10^6^ and 1.8 × 10^6^ TCID_50_/ml for Ac-T7-BrCr and Ac-T7-GFP-BrCr, respectively. To further confirm the recovered EV71, an immunofluorescence assay was performed to analyze the expression of viral structural protein VP1. The immunofluorescence assay results showed that, consistent with the VP1 expression of WT BrCr, the VP1 of recovered viruses was also detected (Supplementary Figure 1).

### Confirmation of Enterovirus 71 Reconstruction in Cell Culture

**FIGURE 3:**
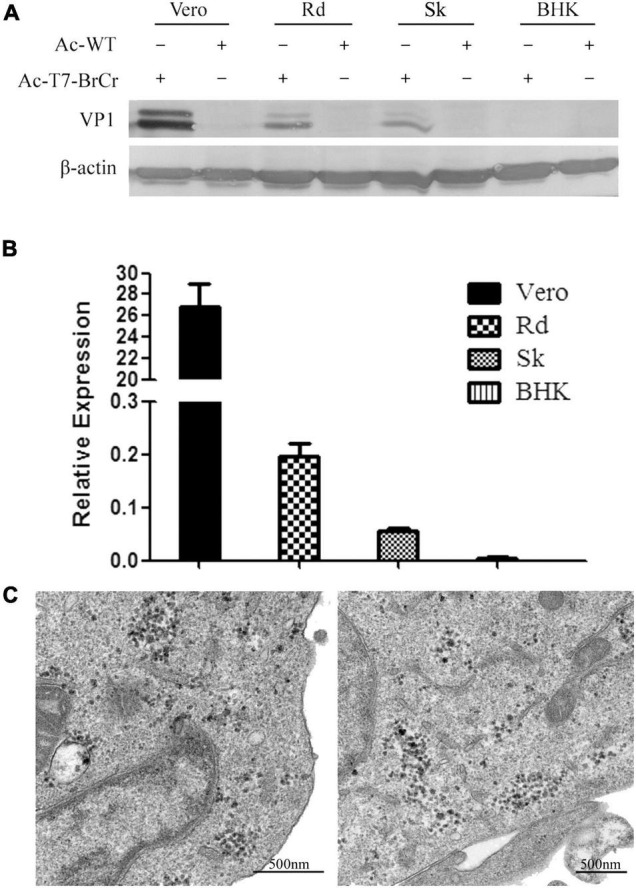
Confirmation of EV71 reconstruction in cell culture. **(A)** Expression of VP1 in mammalian cells transduced with vAc-T7-BrCr. Cells were harvested at 48 h post-transduction, subjected to 10% SDS–PAGE, and immunostained with anti-EV71-VP1 (upper panel) or anti-β-actin (lower panel) antibody. **(B)** mRNA analysis of *Vp1* by real-time PCR after transduction with vAc-T7-BrCr in Vero, Rd, Sk, and BHK cells. All data are represented as the mean ± standard deviation of a set of triplicates. **(C)** Transmission electron microscopy images of the transduced cells. Thirty-six hours after transduction with vAc-T7-BrCr, Vero (left) and Rd cells (right) were fixed and examined for rescued virus. Viruses with a diameter of approximately 30 nm were clearly observed in the cytoplasm of the cells.

To confirm the EV71 recovery, we performed Western blotting to examine the expression of VP1, the major structural protein for EV71 in several mammalian cells (Figure 3A). Forty-eight hours posttransduction with Ac-T7-BrCr or AcMNPV at an MOI of 50, the debris of Vero, Rd, Sk, and BHK cells was collected for analysis. The results showed obvious signals in the Vero, Rd, and Sk cells, while an indistinguishable band was observed in the BHK cells. The cells transduced with wild-type AcMNPV were also analyzed as a negative control.

The generation of viral RNA was subsequently confirmed by real-time PCR. Consistent with the Western blotting results, as shown in Figure 3B, viral RNA in Vero cells transduced with Ac-T7-BrCr was clearly detected and was 10 times higher than that in Rd cells, but the other two cell lines showed detectable levels. This result revealed that recombinant baculovirus delivered the genome to the mammalian cells, but the efficiencies in these three EV71 susceptible cells were different.

Observation of viruses by electron microscopy was a direct evidence for rescued viruses. Therefore, Vero and Rd cells were examined using electron microscopy after transduction with baculovirus Ac-T7-BrCr. TEM images showed that large scales of viruses were in the cytoplasm and had a diameter of approximately 30 nm (Figure 3C), which was not observed in the control group that was transduced with wild-type baculovirus ACMNPV (Supplementary Figure 2).

### Characterization of Recombinant Enterovirus 71 Rescued From the Baculovirus System

**FIGURE 4:**
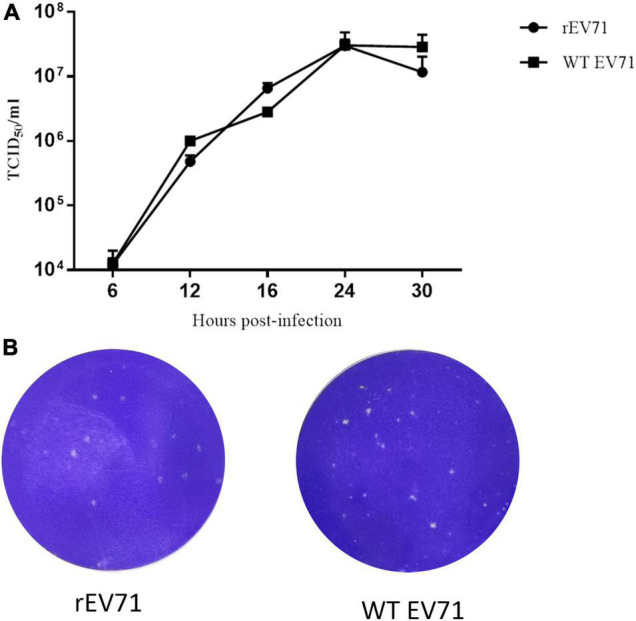
Characterization of rescued EV71. **(A)** Viral growth kinetics of rEV71 and WT EV71. **(B)** Plaque assays of rEV71 and WT EV71.

To investigate whether rEV71 could produce infectious viruses, we used one step growth curve and plaque assay. As shown in Figure 4A, rEV71 showed proliferation rates similar to those of WT EV71 when an MOI of 1 was used to infect Rd cells. At 24 h postinoculation, both strains of EV71 reached peak viral titers, which subsequently declined at 30 h postinoculation. Additionally, similar plaque morphology and number were observed between WT EV71 and rEV71 (Figure 4B).

### Rescued Enterovirus 71 Identification *in vivo*

**FIGURE 5:**
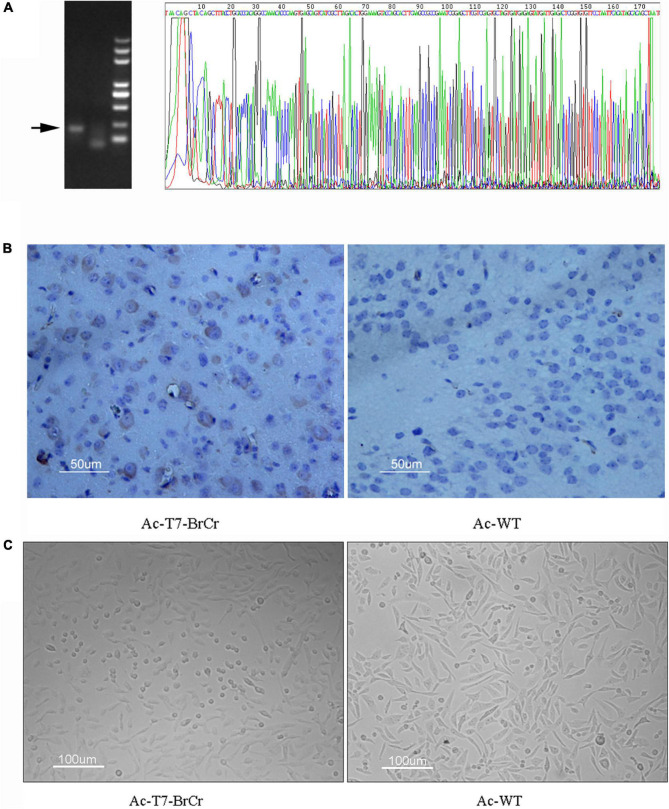
Virus identification of inoculated mice. **(A)** Amplification and sequence confirmation of *Vp1* from brain tissue by reverse transcription polymerase chain reaction (RT-PCR). **(B)** Immunohistochemical expression and localization of VP1. Numerous VP1 protein inclusions were observed in the cytoplasm. **(C)** Reinoculation of purified tissue supernatant into Rd cells and CPE appeared 36 h postinoculation.

To identify the reconstruction of EV71 *in vivo*, we intracranially injected the baculoviruses Ac-T7-BrCr and AvMNPV into 1-day-old ICR mice. The transcription and translation of VP1 were detected by RT-PCR and immunohistochemistry methods 7 days after inoculation. A definite fragment was amplified from brain samples by RT-PCR, and the sequence was exactly identical to that of the parental virus (Figure 5A). Additionally, the immunohistochemical expression and localization of VP1 were noted in brown for tissues from the brains injected with Ac-T7-BrCr, while no signal was observed in the control group, which indicated the existence of the rescued virus (Figure 5B). Furthermore, at 36 h postincubation of purified supernatant from mouse brains with Rd cells, an obvious CPE was detected, which further demonstrated the infectivity of the rescued virus (Figure 5C).

## Discussion

The baculovirus expression system has been extensively applied not only for eukaryotic protein expression in insect cells but also for gene delivery to vertebrate cells, even primary and stem cells (Boyce and Bucher, 1996; Jarvis, 2009). Because of its high capacity for DNA, high transduction efficiency, and biosafety, this system has also been widely used for biotechnology, gene therapy, and vaccine research (Kost et al., 2005). Additionally, a lack of replication ability and low toxicity are the advantageous features for gene expression compared with the widely used vaccinia-T7 polymerase system (Fuerst et al., 1986).

Enterovirus 71 is one of the most common enteroviruses that cause hand–foot–mouth disease (HFMD) and even fatal encephalitis in young children. In addition to the development of antiviral treatment strategies, the major research focus is revealing the mechanisms of EV71 pathogenicity and fundamental questions in EV71 molecular biology and replication. Thus, the availability of full-length genomic cDNA clones for functional analysis of EV71 is a requirement. Furthermore, construction and characterization of an infectious cDNA clone of EV71 will contribute to research on the virus–host interaction, particularly when the virus does not have an efficient *in vitro* replication system (Delaney and Isom, 1998; Arita et al., 2005).

For the generation of infectious virus, the transcription plasmids for full-length cDNA clone of EV71 were linearized, followed by *in vitro* transcription by T7 RNA polymerase, and then the infectious RNA transcripts were purified and transfected into the indicated cells. However, the efficiency of transcription and RNA stability are possible obstacles for rescue of viruses. In this research, EV71 cDNA and the T7 RNA polymerase gene were cloned in one recombinant baculovirus (Figure 1), which strongly improved the efficiency of entering target cells. After transduction in mammalian cells, typical CPEs, such as cell rounding, fall off, floatation, etc., or fluorescence signals were visualized in the cells transduced with recombinant baculoviruses (Figure 2). Furthermore, we observed virus particles of approximately 30 nm located in the cytoplasm, which could not been seen in the control group transduced with wild-type baculovirus AcMNPV (Supplementary Figure 2), in Rd and Vero cells by electron microscopy. Then one-step growth curve and plaque assay were administrated to understand the infectivity of the progeny virus. Our results indicated that infectious EV71 was recovered and had similar growth kinetics and plaque morphology with wild-type EV71 (Figure 4). These results indicated that, compared with other strategies (Arita et al., 2005; Zhang et al., 2020; Yang et al., 2021), EV71 was rescued by a baculovirus expression system and displayed characteristics similar to those of the parental strain. Notably, this rescue system that delivered EV71 cDNA and the T7 RNA polymerase gene together by transduction into mammalian cells eliminated the requirement of additional T7 RNA polymerase and *in vitro* RNA synthesis, which increased the efficiency and cost savings of reverse genetics systems for basic research.

Baculoviruses with mammalian promoters have been harnessed for efficient gene delivery to mammalian cells and enter non-permissive cells for gene delivery (Mansouri et al., 2016; Ono et al., 2018; Hu et al., 2019). For some viruses that have a strict virus–host range, the major hurdles in research are no cell model systems *in vitro* and *in vivo*. For example, recombinant baculovirus was applied to HBV, and consistent HBV genes and infectious virions were observed upon transduction to liver cell lines (Delaney and Isom, 1998; Lucifora et al., 2008). Previous studies have reported that baculovirus systems can rescue viruses in mammalian cells, but the biological characteristics of the rescued viruses depend on themselves and are not related to baculoviruses (Yap et al., 1997). Here, we constructed EV71 infectious clones with a baculovirus system and detected the gene expression of rescued virus in different cells. The accumulation of viral RNA and protein expression of VP1 in Vero, Rd, Sk, and BHK cells after transduction with 50 MOI of baculovirus Ac-T7-BrCr were observed. The results showed a positive signal that varied in different target cells, whereas there was no signal in BHK (Figures 3A,B). Kost et al. (2005) reported that a defect in nuclear transport in certain cell types may block efficient baculovirus-mediated gene delivery. Therefore, the susceptibility to baculovirus transduction or the discrepancy in expression levels that differ in mammalian cells need to be further investigated. In line with previous studies, our results showed that Vero, Rd, and Sk cells were permissive for EV71 replication but not BHK cells (Yamayoshi et al., 2009), although these four cell lines were efficiently transduced by baculovirus (Liang et al., 2004). This result suggests that even though baculovirus has a high transduction efficiency of BHK, infectious EV71 cannot be obtained because of the lack of cell-to-cell spread; in a sense, this baculovirus system with broad host ranges may allow screening of permissive cells for viruses without cell culture or animal models.

In addition to *in vitro* gene expression, baculovirus has been widely used for gene delivery *in vivo*. Sarkis et al. (2000) reported that GFP was expressed in neural cells of mouse and rat brains after injection with baculovirus containing the GFP gene under the control of the cytomegalovirus promoter. Lehtolainen et al. (2002) showed unmodified baculovirus choroid plexus cells in rat ventricles. Hideki et al. also demonstrated that recombinant baculoviruses could efficiently transfer reporter genes not only into primary neural cells *in vitro* but also into the cerebrums of mice *in vivo* (Tani et al., 2003). It has been reported that complement restricts the baculovirus vector transcription when it is injected intravenously or directly into the liver (Sandig et al., 1996). Interestingly, infectious EV71 was successfully produced in an *in vivo* model by the baculovirus delivery system in our study (Figure 5). To the best of our knowledge, this is the first report that EV71 recombinant baculovirus can be delivered to, and replicate in, neurocyte after intracerebral injection of purified recombinant baculovirus (Figure 5B). The *Vp1* gene and protein from the mouse brain were detected by RT-PCR and immunohistochemistry (Figure 5A). Moreover, a typical CPE was observed after inoculation of the supernatant from mouse brain with Rd cells (Figure 5C). All these results indicated that we successfully reconstructed EV71 by a baculovirus delivery system *in vivo*, which can provide a safe and convenient platform for the exploration of animal models and vaccines (Sarkis et al., 2000; Lehtolainen et al., 2002; Tani et al., 2003; Zhang et al., 2020).

In conclusion, we established a novel approach to rescue EV71 *in vitro* and *in vivo* based on a baculovirus expression system, which may provide with a safe and convenient platform for fundamental research and a strategy to rescue viruses that currently lack suitable cell culture and animal models.

## Data Availability Statement

The datasets presented in this study can be found in online repositories. The names of the repository/repositories and accession number(s) can be found in the article/Supplementary Material.

## Ethics Statement

The animal study was reviewed and approved by (No: WIVA07201502) the Institutional Animal Care and Use Committee of Wuhan Institute of Virology, Chinese Academy of Sciences.

## Author Contributions

HW, BLi, and LW were responsible for the design of the work. BLu and QT performed and drafted the manuscript. QT, QW, XL, ZL, and HP were responsible for the literature review, data analysis, and revision of the manuscript. All authors contributed to the article, read, and approved the final manuscript.

## Conflict of Interest

The authors declare that the research was conducted in the absence of any commercial or financial relationships that could be construed as a potential conflict of interest.

## Publisher’s Note

All claims expressed in this article are solely those of the authors and do not necessarily represent those of their affiliated organizations, or those of the publisher, the editors and the reviewers. Any product that may be evaluated in this article, or claim that may be made by its manufacturer, is not guaranteed or endorsed by the publisher.

## Supplementary Material

Supplementary Figure 1 Immunofluorescence assay of rescued EV71 in Vero cells. Immunofluorescence assay was carried out to test the expression of viral structural protein VP1 (red) after transducting Vero cells with wide type AcMNPV (left), v-Ac-T7-BrCr (middle), and v-T7-GFP-BrCr (right).

Supplementary Figure 2 Transmission electron microscopy images of Vero (left) and Rd (right) cells that transduced with the wide type baculovirus AcMNPV control.
