# Supplementary figures and images for: Recovery Infectious Enterovirus 71 by Bac-to-Bac Expression System in vitro and in vivo
Source: Front Microbiol. 2022 Feb 25;13:825111. doi: 10.3389/fmicb.2022.825111 (PMC8959925; doi:10.3389/fmicb.2022.825111)

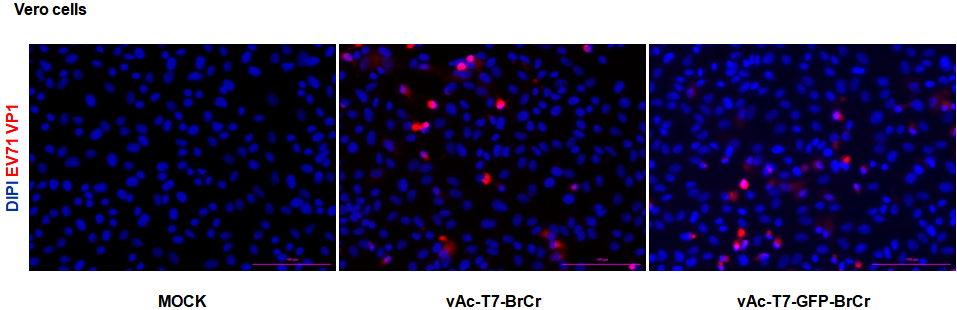

Supplement: Supplementary Figure 1 — Immunofluorescence assay of rescued EV71 in Vero cells. Immunofluorescence assay was carried out to test the expression of viral structural protein VP1 (red) after transducting Vero cells with wide type AcMNPV (left), v-Ac-T7-BrCr (middle), and v-T7-GFP-BrCr (right). [file Image_1.TIF]

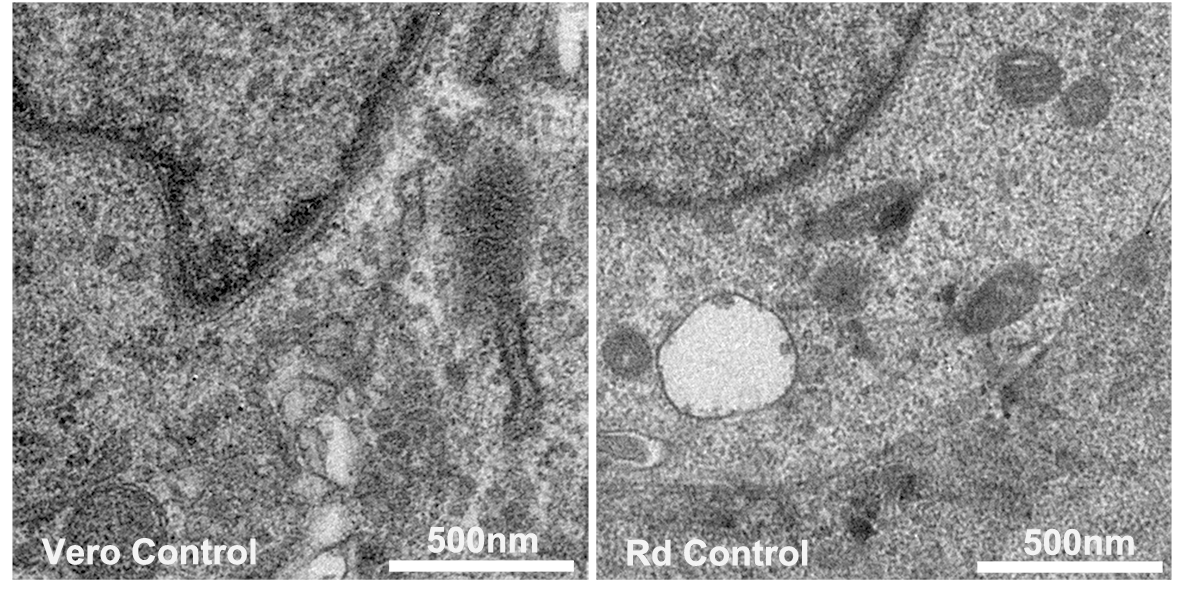

Supplement: Supplementary Figure 2 — Transmission electron microscopy images of Vero (left) and Rd (right) cells that transduced with the wide type baculovirus AcMNPV control. [file Image_2.TIF]
